# Presence or Absence of *mlr* Genes and Nutrient Concentrations Co-Determine the Microcystin Biodegradation Efficiency of a Natural Bacterial Community

**DOI:** 10.3390/toxins8110318

**Published:** 2016-11-03

**Authors:** María Ángeles Lezcano, Jesús Morón-López, Ramsy Agha, Isabel López-Heras, Leonor Nozal, Antonio Quesada, Rehab El-Shehawy

**Affiliations:** 1 IMDEA Water Institute, Alcalá de Henares, Madrid 28805, Spain; angeles.lezcano@imdea.org (M.Á.L.); jesus.moron@imdea.org (J.M.-L.); isabel.lopez@imdea.org (I.L.-H.); leonor.nozal@imdea.org (L.N.); 2 Leibniz Institute of Freshwater Ecology and Inland Fisheries, Berlin 12587, Germany; agha@igb-berlin.de; 3 Departamento de Biología, Universidad Autónoma de Madrid, Cantoblanco 28049, Spain; antonio.quesada@uam.es

**Keywords:** microcystin, biodegradation, cyanobacteria, genotype, *Sphingopyxis* sp., *Paucibacter* sp.

## Abstract

The microcystin biodegradation potential of a natural bacterial community coexisting with a toxic cyanobacterial bloom was investigated in a water reservoir from central Spain. The biodegradation capacity was confirmed in all samples during the bloom and an increase of *mlr*A gene copies was found with increasing microcystin concentrations. Among the 24 microcystin degrading strains isolated from the bacterial community, only 28% showed presence of *mlr*A gene, strongly supporting the existence and abundance of alternative microcystin degradation pathways in nature. In vitro degradation assays with both *mlr*^+^ and *mlr*^−^ bacterial genotypes (with presence and absence of the complete *mlr* gene cluster, respectively) were performed with four isolated strains (*Sphingopyxis* sp. IM-1, IM-2 and IM-3; *Paucibacter toxinivorans* IM-4) and two bacterial degraders from the culture collection (*Sphingosinicella microcystinivorans* Y2; *Paucibacter toxinivorans* 2C20). Differences in microcystin degradation efficiencies between genotypes were found under different total organic carbon and total nitrogen concentrations. While *mlr*^+^ strains significantly improved microcystin degradation rates when exposed to other carbon and nitrogen sources, *mlr*^−^ strains showed lower degradation efficiencies. This suggests that the presence of alternative carbon and nitrogen sources possibly competes with microcystins and impairs putative non-*mlr* microcystin degradation pathways. Considering the abundance of the *mlr*^−^ bacterial population and the increasing frequency of eutrophic conditions in aquatic systems, further research on the diversity of this population and the characterization and conditions affecting non-*mlr* degradation pathways deserves special attention.

## 1. Introduction

Mass developments of cyanobacteria in freshwater systems are increasing worldwide due to anthropogenic eutrophication and global warming [1,2,3]. Some cyanobacterial genera are known to synthesize toxic secondary metabolites, so-called cyanotoxins, which cause human health problems and pose ecological risks [4,5,6,7]. The most widespread and frequently found cyanotoxins are microcystins (hereafter MCs), a group of potent hepatotoxins produced by the genera *Microcystis*, *Dolichospermum*, *Planktothrix*, *Aphanizomenon*, *Nostoc*, and *Anabaenopsis* [8,9]. MCs act as an inhibitor of protein phosphatases 1 and 2A and are, therefore, toxic to eukaryotic cells [10,11]. MCs are heptapeptides synthesized non-ribosomally by large multi-enzyme complexes encoded by the *mcy*A-J gene cluster [12], which serves for the biosynthesis of the complete MC molecule by joining each amino acid. Due to variable incorporation of amino acids MCs presents over 90 different variants [6,13]. 

MCs are structurally stable and resistant against physical and chemical processes such as high temperatures, extreme pH, sunlight, general hydrolytic enzymes, etc. [14,15]. However, some naturally occurring bacterial populations are reported to effectively degrade MCs [16,17,18]. A MC-LR biodegradation pathway has been characterized in some bacterial strains that involve the action of three specific peptidases and a putative oligopeptide transporter encoded in the *mlr*A-D gene cluster [19]. The first enzyme encoded by the *mlr*A gene cleaves the Adda-Arg peptide bond from the MC cyclic structure, resulting in a linearized molecule that is 160 times less toxic [20]. Therefore, the *mlr*A gene has been considered to be an important marker for the detection of bacterial populations with MCs degradation capacity [21,22]. However, the absence of *mlr* genes (or no PCR amplification) has been reported in some MCs degraders, suggesting the existence of alternative degradation pathways [23,24]. A large array of MCs-degrading bacteria, both containing and lacking *mlr* genes, have been isolated from both water and sediment samples. Most of these strains belong to the phyla Proteobacteria (especially classes α- and β-Proteobacteria) [25,26,27], although strains from the phyla Actinobacteria and Firmicutes have also been reported [23,28].

The study of MCs degradation kinetics of indigenous bacteria can increase understanding of the fate and lifetimes of MCs in the water column. Although substrate competition with other organic and inorganic compounds in natural waters appears to play a crucial role in the MCs degradation process [29], no studies have been performed to study the effect of nutrient availability on the MCs biodegradation efficiency between *mlr*^+^ and *mlr*^−^ bacterial genotypes (with presence and absence of the *mlr* gene cluster, respectively). Few studies have focused on comparing MCs degradation rates in bacterial strains under different nutrient conditions, and those they have been carried out report contradictory results. Studies performed directly on biofilm communities showed lower MC-LR degradation removal rates in media spiked with acetate [30], glucose and peptone [31], but enhanced rates with the addition of nitrate [31]. On the other hand, studies developed with individual strains found that the presence of exogenous C and/or N sources enhanced MC-LR removal rates in some cases [32,33] and impaired them in others [30]. Unraveling the effect of nutrients in MCs biodegradation is especially relevant after a bloom collapse and sestonic MCs are released, since cell debris and other nutrients from the water column may serve as alternative C and N sources. In this sense, *mlr*^+^ and *mlr*^−^ genotypes from the natural MCs-degrading bacterial community may respond differently in terms of degradation efficiency. To address this, field and experimental approaches with a natural bacterial community and isolates from said community were used. 

This study aims to analyze the diversity among strains of a natural bacterial community in relation to the presence/absence of *mlr* genes and their MCs degradation efficiencies. In particular, the objectives of the present work are: (i) to evaluate the MCs biodegradation capacity of a natural bacterial community from a water body in the Iberian Peninsula (Mediterranean region) and isolate the responsible bacterial strains; and (ii) to study the MCs biodegradation efficiency of both *mlr*^+^ and *mlr*^−^ bacterial genotypes under variable total organic carbon and total nitrogen concentrations. 

## 2. Results

### 2.1. Cyanobacterial Bloom and Biodegradation Capacity of the Natural Bacterial Community

The presence of potentially MCs-producing cyanobacteria along the bloom episode was recorded in all samples by microscopic identification [34]. *Microcystis aeruginosa* was the dominant species along the bloom period, but *Microcystis flos-aquae*, *Microcystis wessenbergii* (non-toxic), *Dolichospermum crassa*, *Woronichinia naegeliana* and *Aphanizomenon flos-aquae* were also identified. MCs-producing cyanobacteria were also confirmed by the presence of *mcy*E gene in all samples, used in this study as a marker for potentially MCs-producing cyanobacteria. Total sestonic MCs ranged from 0.54 to 49.52 µg·L^−1^, peaking on 24 September (Table 1). MC-LR was the most abundant variant in all samples, representing approximately the 59% (±8%) from total MCs, followed by MC-RR, with 29% (±5%) and MC-YR, with 12% (±3%). In order to assess the MCs degradation capacity of the natural bacterial community at different bloom stages, water samples were spiked with 1.2 mg equivalent MC-LR·L^−1^ of crude MCs. After 15 days of incubation all samples showed complete removal of MCs (Table 2), evidencing an endogenous MCs degradation capacity during the bloom episode. 

### 2.2. Isolation of MCs-Degrading Bacteria from the Bacterial Community

**Figure 1:**
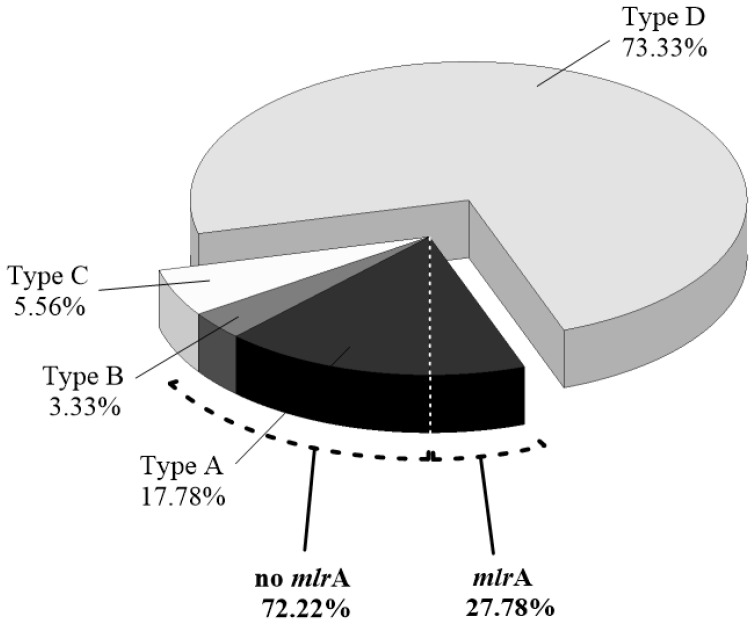
Proportion of the different cultivable MCs-degrading bacterial phenotypes found in the water samples. Classification was performed according to MCs degradation efficiencies. Type A represents bacteria with a MCs degradation efficiency between 80% and 100%, type B between 60% and 79% and type C between 40% and 59%. Non-MCs-degrading bacteria are represented in type D. Proportion of the most efficient MCs-degrading bacteria (phenotypes A and B) with presence and absence of *mlr*A gene are also represented.

In order to characterize the natural MCs-degrading bacterial community, 90 bacterial strains were isolated from the MCs enrichment assay. Using six-day in vitro degradation assays in ¼ R2A medium enriched with 1 mg equivalent MC-LR·L^−1^ of raw MCs, individual isolates were classified into four phenotypes according to their MCs degradation efficiencies (Figure 1). Phenotype A comprises the most efficient MCs-degrading group of bacteria with a degradation efficiency ranging between 80% and 100%, followed by phenotype B and C, with efficiencies ranging between 60% and 79%, and 40% and 59%, respectively. Phenotype D comprises non-MC-degraders and represents the largest group, with the 73.33% of the total bacterial isolates (66 strains), followed by phenotype A, C and B, represented by the 17.78% (16 strains), 5.56% (5 strains) and 3.33% (3 strains), respectively. Among the 24 MCs-degrading bacterial isolates belonging to phenotypes A, B and C (mostly isolated on 4 September), only the 19 strains from the most efficient phenotypes (A and B) were screened for the presence of *mlr*A gene, revealing that *mlr*A was only present in a relatively small fraction of the MCs-degrading strains investigated (27.78%). 

### 2.3. Abundance of mlrA Gene during the Bloom Episode 

**Figure 2:**
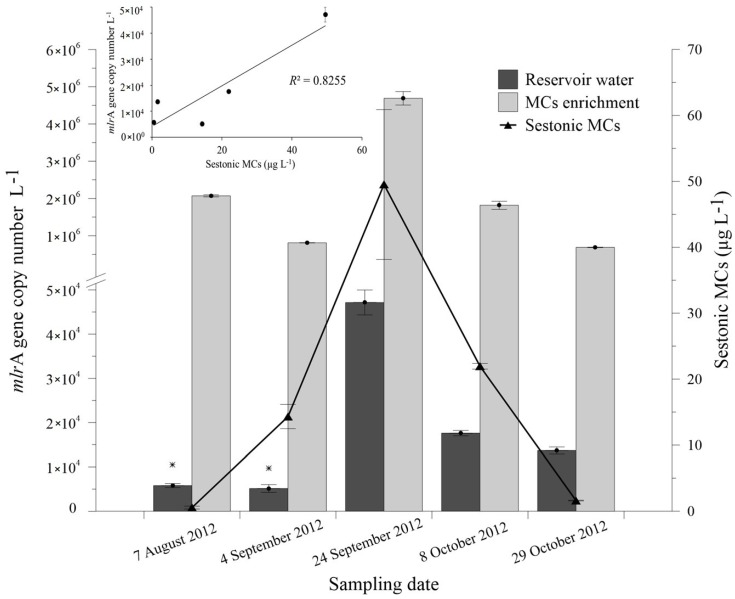
Abundance of *mlr*A gene copies L^−1^ in reservoir water samples and 15 days after MCs enrichment. Line represents total sestonic MCs and the small inserted plot represents the correlation between *mlr*A gene copies L^−1^ and total sestonic MCs. Asterisk indicates data below quantification limit and error bars represent standard errors of two replicates.

Quantitative analysis of the *mlr*A gene by Real-Time PCR during the bloom episode ranged from unquantifiable (but detected) gene copies L^−1^ on 7 August and 4 September, to 4.72 × 10^4^ gene copies L^−1^ on 24 September, 1.76 × 10^4^ gene copies L^−1^ on 8 October and 1.37 × 10^4^ gene copies L^−1^ on 29 October (Figure 2). These results, together with the high correlation recorded between *mlr*A gene abundance and sestonic MCs, show that *mlr*A^+^ bacterial population (with presence of *mlr*A gene) follows a similar trend with total sestonic MCs concentrations, peaking on 24 September, when maximum MCs concentration was also recorded. Quantitative analysis of the *mlr*A gene in reservoir water spiked with 1.2 mg equivalent MC-LR·L^−1^ of crude MCs showed an increase of gene abundance near two orders of magnitude in all samples, indicating a positive response of *mlr*A^+^ bacterial population to MCs concentrations in the water. 

### 2.4. In Vitro MCs Biodegradation Assays and Kinetics

**Figure 3:**
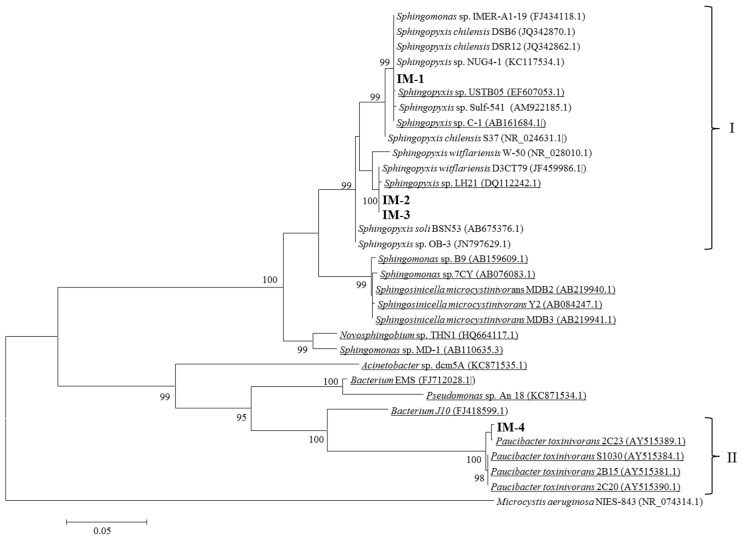
Maximum likelihood tree based on the 16S rRNA gene (1344–1420 bp) showing, in bold, the position of the sequences obtained in the present study. The numbers near nodes indicate bootstrap values greater than or equal to 95, as a percentage of 1000 replicates resulting from the analysis. Underlined sequences indicate already known MCs-degrading bacteria. Bar, 0.05 substitutions per nucleotide position. Cluster I represents *Sphingopyxis* sp. and cluster II, *Paucibacter toxinivorans*.

In order to evaluate whether the presence or absence of *mlr* genes determine MCs degradation efficiencies under alternative carbon and nitrogen sources, four new isolates and two MCs-degrading bacteria from culture collection (*Sphingosinicella microcystinivorans*, strain Y2 and *Paucibacter toxinivorans*, strain 2C20) were selected. Results from the identification of the complete *mlr* gene cluster showed that the new isolates IM-1, IM-2 and IM-3, identified as *Sphingopyxis* sp. based on 16S rRNA analysis (Figure 3), contain the whole *mlr* cluster (*mlr*A-D)(*mlr*^+^ genotype) while strain IM-4, identified as *Paucibacter toxinivorans* (Figure 3), lack the complete *mlr* gene cluster (*mlr*^−^ genotype). Bacterial strains Y2 and 2C20 were identified as *mlr*^+^ and *ml*r^−^ genotypes, respectively. 

**Figure 4:**
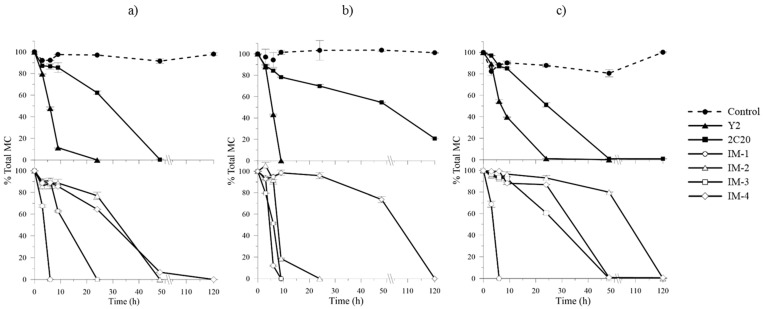
Biodegradation of total MCs by bacterial genotypes *mlr*^+^ (strains Y2, IM-1, IM2 and IM-3) and *mlr*^−^ (strains 2C20 and IM-4) incubated in: (**a**) MSM; (**b**) ¼ R2A medium; and (**c**) reservoir water for 120 h. Upper plots represent bacteria from culture collection and bottom plots represent new isolated strains. A negative control without bacteria was included. Error bars represent standard errors of two technical replicates.

MCs degradation kinetics of strains were assessed under different concentrations of alternative carbon and nitrogen sources by using Minimal Salts Medium [26] (absence of carbon and nitrogen compounds) and ¼ R2A medium (258.37 ± 0.06 mg·L^−1^ of TOC and 1.9 mg·L^−1^ of TN). An experiment in reservoir water (3.83 ± 0.02 mg·L^−1^ of TOC and <0.05 mg·L^−1^ of TN) was also performed to evaluate degradation efficiencies similar to real life scenarios. Experiments were performed with 1 mg equivalent MC-LR·L^−1^ of a MCs crude extract containing 92.37% (±0.86%) of MC-LR, 3.91% (±0.82%) of MC-RR and 3.71% (±0.50%) of MC-YR. Figure 4 shows MCs degradation curves of selected strains over time. In all cases, no losses of MCs were found in negative controls, indicating that removal of MCs was biologically-mediated. *mlr*^+^ bacterial genotype (strains IM-1, IM-2, IM-3 and Y2) considerably improved MCs degradation and reduced lag phases under high carbon and nitrogen concentrations (¼ R2A medium) compared to MSM and reservoir water. Contrarily, *mlr*^−^ genotype (strains 2C20 and IM-4) showed longer lag phases and required 120 h or more to completely degrade the toxins in presence of carbon and nitrogen sources. Only strain IM-1 (*mlr*^+^) was equally efficient degrading 90% of total MCs in 6 h regardless of the different TOC and TN concentrations. MCs degradation rates in ¼ R2A medium comparing to MSM was enhanced about 1.5, 3.8 and 3.0 times in Y2, IM-2 and IM-3, respectively (*mlr*^+^), and was depressed about 1.6 and 4.7 times in 2C20 and IM-4, respectively (*mlr*^−^) (Table 3). Regardless of the genotype, no bacterial growth was observed either in MSM medium or in reservoir water, where no or few carbon and nitrogen sources apart from MCs (and other natural components of the MC raw extract) were present (Table S1 from Supplementary Materials).

Individual analysis of MC-LR and MC-RR biodegradation in selected bacterial strains showed no apparent differences in degradation rates under above-mentioned testing conditions (Figures S1–S3 from Supplementary Materials). Data from MC-YR were excluded from this analysis because of discrepancies among technical replicates due to its low sensitivity and low initial concentration.

## 3. Discussion

In the current study, all samples, except those collected on 7 August, exceeded the provisional drinking water guideline value of 1 µg·L^−1^ for MCs [35]. Although no guideline value for MCs is reported for recreational waters, the WHO establishes a maximum of 20,000 cyanobacterial cells mL^−1^ or 10 µg·L^−1^ of chlorophyll a for low health effects, where about 2–4 µg·L^−1^ of MCs is expected [36]. Accordingly, samples collected on 4 September, 24 September and 8 October exceeded these values. These MCs concentrations were within the range or higher than those reported in previous studies that were conducted on water samples taken in Spain [37,38,39], Europe [40,41,42] and other countries [43,44], which questions the capacity of the ecosystem to tackle high concentrations of MCs and, thus, prevent their potential harmful effects on humans, animals and other living beings. The depletion of MCs in spiked reservoir water samples collected during the bloom episode revealed the MCs biodegradation capacity of the indigenous bacterial community to overcome a release of intracellular MCs during bloom decay. This capacity may also explain, together with photodegradation, adsorption and/or dilution factors, why the extracellular fraction is always several orders of magnitude below the sestonic [8]. Considering predictive models of climate change, temperate lakes are expected to extend periods of thermal stratification under warmer conditions, lengthening growing season of primary producers and uncoupling trophic relationships [2,45]. This, in addition to expected decreases in precipitation in the Mediterranean area, will lead to longer water residence times resulting in higher total cyanobacterial biomass and increased cyanotoxin concentrations in water [46]. In such projected scenarios, the endogenous microcystin biodegradation process in Mediterranean reservoirs and lakes will become an important mechanism for restoration of initial conditions and water uses. Thus, the study of said mechanism and the factors controlling the efficiency of the process in nature deserves special attention. 

Coexisting MCs-degrading strains in the bacterial community were classified according to their different degradation efficiencies into three phenotypes (A–C). Thus far, most studies have been mainly focused on isolation and simple identification of new MCs-degrading strains. However, the screening and classification of coexisting bacteria according to their degradation efficiencies has allowed understanding that the composition of these phenotypes within the indigenous bacterial community may modulate and ultimately be responsible for the final MCs concentration in nature when a cyanobacterial bloom develops. Previous studies on cultivable heterotrophic bacteria have demonstrated the coexistence of different bacterial genera associated with cyanobacterial blooms [47], and identified qualitative and quantitative family composition changes in relation to the amount of cyanobacterial biomass [48]. These results support our observations of the existence of diverse MCs-degrading bacteria with different degradation efficiencies, and that the occurrence, proportion, and spatio-temporal distribution of these MCs-degrading phenotypes contribute to the regulation of the final concentration of MCs in the water. The presence of two different genotypes, *mlr*A^+^ and *mlr*A^−^, within the MCs-degrading bacterial community support previous studies that suggest the existence of alternative pathways for the MCs degradation process to that described by Bourne et al. (2001) [19,23,24]. Another possibility is that, contrary to the general consensus in the field, *mlr*A gene is not highly conserved among the genera capable of degrading MCs. This, in addition to the lack of *mlr*A gene analysis of some novel MCs-degrading bacteria [18], makes it difficult to establish a firm conclusion about the suitability of the *mlr*A as an unequivocal gene marker for MCs-degrading capacity. According to our results, in vitro MCs biodegradation assays currently represent the most reliable test to assess the MCs-degrading ability of a strain. The design of universal primers or the elucidation of new enzymatic pathways may reveal new insights into the MCs degradation process and the biological role these pathways play in the removal of MCs in nature. 

The observed temporal variations in *mlr*A gene abundance during bloom development, together with the positive response of *mlr*A to enriched water samples with crude MCs, revealed that the *mlr*A^+^ bacterial population responded positively to a release of MCs in water. Similar results were observed by Zhu et al. (2014) [49], who found that seasonal variations of extracellular MCs in water were related to variations in MCs-degrading bacterial abundance based on *mlr*A gene analysis. Our results, together with other studies, suggest that these *mlr*A^+^ bacteria make an important contribution to the biodegradation of MCs in water [49,50]. However, the real contribution of the total biodegradation of MCs in nature can only be understood by considering the whole MCs-degrading bacterial community which, according to our results, goes beyond the analysis of the *mlr*A gene. Therefore, although correlations between *mlr*A gene copy number and MC-LR biodegradation rate identified by Li et al. (2015) [50] exhibited a similar changing trend, the low correlation (*R*^2^ = 0.54) actually indicates that biodegradation rate of MCs is only partially explained by *mlr*A-possessing MCs-degrading bacteria. This is consistent with the relatively higher abundance of *mlr*A^−^ over *mlr*A^+^ MCs-degrading bacteria found in our study and in Mou et al. (2013) [24], which supports our observation that the MCs-degrading bacterial population that lacks *mlr* genes may be an important contribution to the MCs biodegradation process in nature. In an effort to better understand the importance and advantage of *mlr* genes for the MCs-degrading bacterial community, we studied the degradation efficiencies of both genotypes (*mlr*^+^ and *mlr*^−^) in presence of various TOC and TN concentrations. 

Our results show dissimilar MCs degradation efficiencies between *mlr*^+^ and *mlr*^−^ bacterial genotypes in presence of alternative C and N sources. The marked improvement of MCs removal rates by genotype *mlr*^+^ under alternative C and N compounds compared to bacteria in absence of *mlr* genes (Figure 4) suggests a different microcystin substrate affinity between *mlr*^+^ and alternative non-*mlr* degradation pathways. According to our results, the presence of alternative organic carbon and nitrogen (in this study provided by ¼ R2A medium) stimulate both growth (Table S1 from Supplementary Materials) and MCs degradation in *mlr*^+^ strains (Figure 4), indicating that MCs removal rates seem to depend more on the abundance of bacterial biomass, as reported in other studies [21,51] than on the stimulation or depression of specific nutrients to *mlr* genes expression [52]. Our results are also in accordance with Zhang et al. (2015) [32], where removal percentage of MC-LR increased by increasing bacterial growth under addition of glucose and ammonium chloride. In contrast, despite showing growth of *mlr*^−^ strains in presence of alternative C and N sources, MCs degradation was not enhanced, indicating a possible competition between MCs and alternative substrates. Accordingly, the absence of *mlr* genes and, consequently, the presence of alternative MCs degradation pathways [23,24], drives lower degradation rates in presence of additional C and N sources compared to those containing *mlr* genes. These differences in the degradation efficiency among natural MCs-degrading bacteria raise concern about its impact in the aquatic ecosystem, where alternative C and N sources are always present in the water column. The low C and N concentrations found in the reservoir water we used in this study explain why MCs degradation rates are similar to those obtained in absence of alternative C and N sources (MSM). However, the increasing eutrophication of aquatic ecosystems raises doubts about the MCs degradation efficiency of the indigenous bacterial community. It appears to us that eutrophication may not only drive more incidences of CyanoHABs, but may also drive lower overall elimination of MCs. Despite the enhanced MCs removal of *mlr*^+^ bacteria when other C and N sources are present in the water, the degradation capacity of the abundant *mlr*^−^ bacterial population is negatively affected under increasing C and N concentrations. This, in turn, may result in prolonged toxicity of the bloom episode. 

The equal degradation of both MC-LR and MC-RR by our isolated strains in the presence of variable C and N concentrations (Figures S1–S3) indicates that both *mlr* and alternative non-*mlr* pathways do not appear to exhibit preference among MC variants, which is consistent with other studies where no differences were found either in reservoir water samples [53] or in mineral salts medium [50]. Therefore, regardless of common shifts in the relative abundance of MC variants along the bloom episode, MC degraders from the bacterial community will face these changes with similar efficiency, as they do not show any preference for one type over the other.

## 4. Conclusions

Our results demonstrate that the endogenous MCs degradation capacity found in an aquatic system during a cyanobacterial bloom is mediated by a MCs-degrading bacterial community and contributes to the natural capacity of the ecosystem to cope with high MCs loads. The presence or absence of the *mlr* gene cluster in the MC-degrading bacterial community has been proven to be an important factor for the MCs degradation efficiency under the presence of alternative carbon and nitrogen sources. Bacteria with *mlr* genes showed better MCs substrate affinity and higher degradation rates than MC-degrading bacteria without *mlr* genes. Accordingly, the MCs removal rates in nature are not only determined by the availability of alternative C and N sources, but also the relative abundance of *mlr*^+^ over *mlr*^−^ genotypes within a MC-degrading bacterial community. Our findings further highlight the need to increase our knowledge on the diversity, abundance and function of the MCs-degrading bacterial community under eutrophication and global warming scenarios in order to predict their impacts on the ecosystem.

## 5. Materials and Methods

### 5.1. Sampling

A permanent pond in Alberche’s river located near the exit of the San Juan dam (Madrid, Spain) (40°22′3.43′′ N and 4°18′12.28′′ W) was sampled from 7 August 2012 to 29 October 2012, comprising a cyanobacterial bloom episode. Samples were taken on a monthly basis from 7 August to 4 September then, collected every two weeks until the end of the sampling period. At every sampling event, a total of 4 L of sub-surface water samples were collected with sterile polyethylene bottles and transported in the dark at 4 °C. One liter of water was sequentially filtered through fiberglass filters (2.7 µm approx., Millipore, Darmstadt, Germany) to remove algae and large cyanobacteria and then through 0.22 µm polycarbonate filters (Millipore) to collect the bacterioplankton. Polycarbonate filters were stored at −20 °C until DNA extraction. Another 1 L was passed through fiberglass filters (0.7 µm approx., Millipore) to collect seston and stored at −20 °C until analysis. Some water aliquots were used immediately after collection to screen for MC-degrading bacteria. Finally, 1 L of water was used for taxonomic identification of buoyant cyanobacteria under microscope (Olympus CX41, Tokyo, Japan) after 24 h of flotation [34].

### 5.2. Microcystins Extraction 

A crude microcystin extract for experiments was extracted from a scum collected in San Juan reservoir. The scum was extracted with 100% methanol, vortexed for 1 min, sonicated for 10 min in an ultrasonication bath (P-Selecta Ultrasons, Barcelona, Spain) and stored at 4 °C for one hour for extraction. The extracted sample was then centrifuged (Sorvall RC-5C, GMI, Ramsey, MN, USA) for 15 min at 4000× *g* and supernatant was stored at −20 °C. The whole extraction process was repeated three times. MCs crude extract was vacuum dried at 40 °C in a rotavapor (Rotavapor-R, Büchi Labortechnik AG, Flawil, Switzerland), resuspended in 10% methanol and bonded to 5 g C18 cartridges (Extrabond C18, 5 g, 20 mL, Sharlab, Barcelona, Spain) for partial purification. Cartridges were activated with 100% methanol, followed by Milli-Q water and equilibrated with 10% methanol. After that, samples were passed through and washed by Milli-Q water and 30% methanol. MCs were eluted in 20 mL of 90% methanol and dried down under vacuum in a multiple evaporator (Heidolph Instruments GmbH & Co.KG, Schwabach, Germany) at 40 °C. Finally, MCs were resuspended in Milli-Q water, passed through sterile syringe 0.22 µm filters (25 mm, Pall Corporation, Port Washington, NY, USA) for sterilization and stored at −20 °C. 

Sestonic microcystins from fiberglass filters were extracted twice by sonication with 90% aqueous methanol and evaporated at 40 °C under vacuum in the multiple evaporator. Final extracts were stored at −20 °C until analysis. 

### 5.3. Reservoir’s MCs-Degradation Capacity 

The determination of the reservoir’s MCs-degradation capacity was performed in duplicates in 20 mL of raw water enriched with 1.2 mg equivalent MC-LR·L^−1^ of crude MCs extract. Flasks were incubated for 15 days at 27 °C with 120 rpm shaking in the dark to prevent photosynthetic growth. Negative controls consisting of autoclaved reservoir water were included. Samples for quantifying MCs (MC-LR, -RR and -YR) were collected at the start and after 15 days of incubation. Additional samples were filtered through 0.22 µm polycarbonate membranes and filters containing retained microorganisms were stored at −20 °C for DNA extraction. 

### 5.4. Screening and Isolation of MCs-Degrading Bacteria

Samples showing degradation activity in the previous experiment were 10-fold serially diluted and transferred to R2A agar (Sigma-Aldrich, St. Louis, MO, USA) plates. Plates were incubated for 7 days at 27 °C in the dark. A total of 90 colonies with different morphologies were selected and purified by streaking on plates. Each isolated colony was transferred individually into R2A liquid medium and incubated under same conditions until late exponential phase. From each bacterial culture, an aliquot was washed and resuspended in 100 µL of ¼ R2A liquid medium with 1 mg equivalent MC-LR·L^−1^ of crude MCs extract to get a final optical density of 0.45 (maximum error ± 0.15) at 600 nm. Aliquots were transferred to sterile 96-well plates containing 150 µL of ¼ R2A liquid medium with 1 mg equivalent MC-LR·L^−1^ of raw MCs extract in each well. Negative controls (no bacterial inoculation) and positive controls inoculated with strains Y2 [54] and 2C20 [55] were also included. Plates were covered with a sterile plastic film and incubated at 27 °C in the dark at 120 rpm for 6 days. Total MCs concentrations were analyzed at start and after 6 days of incubation. Classification of bacterial phenotypes according to MCs degradation efficiencies was performed using an ascending hierarchical classification analysis with XLSTAT software (Barcelona, Span) [56] with a minimum and maximum variability intra- and inter-cluster, respectively. 

### 5.5. MCs Degradation under Different TOC and TN Concentrations 

Four new MCs-degrading isolates were incubated in R2A medium at 27 °C in the dark at 120 rpm for 24 h with initial absorbance of 0.05 measured at 600 nm. Cells in the late exponential phase were centrifuged at 5.000 rpm for 5 min, washed in Mineral Salts Medium (MSM) [26] and incubated in MSM for 14 h at the same conditions to induce nutrient starvation. Cells were then resuspended at a final absorbance of 0.05 at 600 nm (equivalent to 10^8^ CFU mL^−1^, approximately) in various media (MSM, reservoir water and ¼ R2A liquid medium) with different total organic carbon (TOC) and total nitrogen (TN) concentrations, containing 1 mg equivalent MC-LR·L^−1^ of crude MCs extract. Experiments were performed in duplicate. Total organic carbon and total nitrogen concentrations from the three media were analyzed in triplicates in a Total Organic Carbon analyzer (TOC-V CSH, Shimadzu, Kyoto, Japan) and Total Nitrogen kit (range 0.5–15.0 mg/L, Spectroquant, Merck Millipore, Dramstadt, Germany), respectively. Incubation was carried out under previously described conditions for 120 h and samples were collected at different time intervals (0, 3, 6, 9, 24, 48 and 120 h) for quantification of MCs and bacterial biomass. A standard curve of optical density vs. biomass (dry weight) was performed to calculate the bacterial biomass at each time interval and, thus, the average growth rate. 

### 5.6. Analysis of Microcystins

All samples for MCs analysis were filtered through 0.22 µm syringe filters (Acrodisc GHP, Pall Corporation, Port Washington, NY, USA) before analysis and commercial MC-LR, MC-RR and MC-YR pure standards (Sigma-Aldrich, St. Louis, MO, USA) were used for calibration curves. 

Sestonic MCs concentrations from water samples and MCs from the kinetic experiment were measured on a high-performance liquid chromatography (HPLC) system (Agilent series 1100, Agilent Technologies, Santa Clara, CA, USA) coupled to a time-of-flight (TOF) mass spectrometer (Agilent 6230 accurate mass TOF Agilent Technologies, Santa Clara, CA, USA). Chromatographic separation of MC-LR, MC-RR and MC-YR was performed using a Pursuit C18 150 mm × 2 mm column with 3 µm of particle size (Agilent Technologies, Santa Clara, CA, USA) and thermostated at 40 °C. The mobile phase consisted of 0.1% of acetic acid in water (A) and 0.1% of acetic acid in acetonitrile (B) with a flow rate of 0.3 mL/min. Gradient profile started at 30% B, increased to 60% B over 9 min and changed to 100% B over 1 min for cleaning. For re-equilibration of the column, B was reduced to 30% over 1 min and was held for further 6 min. Fifty microliters of each sample was injected. 

MCs concentrations for the rest of experiments were measured on a Varian 500 Ion Trap Mass Spectrometer (Agilent Technologies, Santa Clara, CA, USA) supported by two Varian 212 LC chromatographic pumps (Agilent Technologies, Santa Clara, CA, USA). Chromatographic separation of MC-LR, MC-RR and MC-YR was performed using a Pursuit C18 150 mm × 2 mm column with 3 µm of particle size (Agilent Technologies, Santa Clara, CA, USA) following conditions from Agha et al. (2012) [37]. 

### 5.7. Genomic DNA Extraction

Genomic DNA extraction from filters and bacterial pellets was performed using DNeasy Plant Mini Kit (QIAGEN, Hilden, Germany) following manufacturer’s instructions with several modifications: Filters and pellets were introduced in 2 mL microcentrifuge tubes with addition of the first buffer (Buffer AP1). For fiberglass filters, they were previously cut into two pieces, each acting as a single sample, to avoid excess of fiber filter residues that may interfere with DNA extraction. Immediately, tubes were introduced for 30 s in liquid N_2_ and thawed at room temperature, repeating the process twice. After thawing, cells were disrupted using sterile glass beads (212–300 µm, acid-washed, Sigma Aldrich, St. Louis, MO, USA) and a homogenizer (Precellys, Bertin Technologies, Montigny-le-bretonneux, France) at 5000 r.p.m. for 40 s. RNAse was added to tubes, incubated at 65 °C for 10 min and then centrifuged at 20,000× *g* for 2 min. Supernatants were transferred to new microcentrifuge tubes avoiding filter residues. At this point, manufacturer’s instructions were followed and both tubes belonging to the same glass fiber filter were combined to the same DNeasy Mini spin column. Genomic DNA was dissolved in sterile Milli-Q water and stored at −20 °C until analysis. 

### 5.8. mlrA-D Genes Detection

For the identification of the *mlr* gene cluster, a PCR using the specific primer sets MF/MR [22], mlrBf1/mlrBr1, mlrCf1/mlrCr1 and mlrDf1/mlrDr1 [25] was carried out. Amplifications were performed in 50 µL of total volume containing final concentration of 0.25 µM of each primer, 1× PCR buffer (HotStarTaq Master Mix kit, QIAGEN, Hilden, Germany) and 1 µL of DNA. Amplification of the *mlr*A gene in a PCR thermal cycler (Techne TC-5000, Bibby Scientific, Staffordshire, UK) was performed under the following conditions: initial activation step at 95 °C for 15 min, followed by 35 cycles of 94 °C for 30 s, 57 °C for 40 s and 72 °C for 40 s. Final extension step at 72 °C for 10 min. Amplification of *mlr*B, *mlr*C and *mlr*D genes were performed with an initial activation step at 95 °C for 15 min, followed by 40 cycles of 95 °C for 30 s, 60 °C for 40 s and 72 °C for 40 s. Final extension step was set at 72 °C for 10 min. PCR products were separated on 1.5% agarose gel electrophoresis and visualized on a AlphaImager HP (Alpha Innotech, San Leandro, CA, USA).

### 5.9. mcyE Gene Detection

Genomic DNA extracted from fiberglass filters were used for identification of the *mcy*E gene as a marker for the presence of potentially MCs-producing cyanobacteria. Specific primer set HEPF and HEPR [57] was used for the PCR. Amplification was performed in 10 µL of total volume, containing final concentration of 0.25 µM of each primer, 1× PCR buffer (HotStarTaq Master Mix kit, QIAGEN) and 1 µL of DNA. PCR amplification was performed under the following conditions: initial activation step at 95 °C for 15 min, followed by 35 cycles of 94 °C for 20 s, 52 °C for 30 s and 72 °C for 1 min. Final extension step at 72 °C for 10 min. PCR products of 472 bp were separated by 1.5% agarose gel electrophoresis and visualized on the AlphaImager HP (Alpha Innotech, San Leandro, CA, USA). 

### 5.10. Quantification of mlrA Gene: Real-Time PCR

Serial dilutions of purified PCR product of *mlr*A gene (807 bp) from strain IM-2 were used to generate a standard curve for *mlr*A detection. A specific primer set qmlrAf and qmlrAr, and the TaqMan probe qmlrA-tm with a variation in the quencher [21], were used for quantification on a Real-Time PCR (AB7300, Applied Biosystems, Foster City, CA, USA). Reactions resulted in an amplification of 120 bp product and were carried out in duplicate in 25 µL of volume containing 1× of QuantiTech Probe PCR Master Mix (QIAGEN), 0.4 µM of each primer, 0.2 µM of qmlrA-tm probe and 1 µL of either a DNA standard or sample. Thermal cycling conditions were performed with an initial activation step of 95 °C for 15 min, followed by 45 cycles of denaturation at 94 °C for 15 s and annealing/extension at 62 °C for 1 min. Data were collected at the end of the annealing/extension step. Gene copies per sample were calculated using a standard curve of the target gene copy number vs. threshold cycle (Ct) with a correlation coefficient (*R*^2^) of 0.999 and an efficiency of 85% in a linear range of 3.16 × 10^3^ to 3.16 × 10^9^ *mlr*A gene copy number per sample. Quantification of *mlr*A gene copies per liter were calculated considering the volume filtered. 

### 5.11. Identification of Bacterial Isolates Using 16S rRNA Gene Analysis

Genomic DNA of the new isolated MCs-degrading bacteria was used for 16S rRNA gene analysis. A PCR was performed using two universal primer sets: (1) 27F and 907R; and (2) 533F and 1492R_l [58,59,60]. Amplification was performed in 50 µL of total volume, containing final concentration of 0.25 µM of each primer, 1× PCR buffer (HotStarTaq Master Mix kit, QIAGEN) and 1 µL of DNA. Amplification for both primer sets was performed under the following conditions: initial activation step at 95 °C for 15 min, followed by 35 cycles of 94 °C for 30 s, 55 °C for 45 s and 72 °C for 1 min. Final extension step at 72 °C for 10 min. PCR products were separated by 1.5% agarose gels electrophoresis with bands visualized on the AlphaImager HP. PCR products were purified with QIAquick PCR purification kit (QIAGEN) following manufacturer’s instructions and sent for sequencing to the Molecular Biology Center of the University of Alcalá (Madrid). Nucleotide sequences obtained from both primer sets were combined (1344–1420 bp) and compared with sequence information available in the NCBI using BLASTN. Multiple alignment of the sequences was performed using CLUSTAL W [61] from the current BioEdit software (version 7.2.5, Ibis Biosciences, Carlsbad, CA, USA) [62]. The construction of the phylogenetic tree was performed with MEGA6 software [63] and a tree based on the 16S rRNA gene was constructed using Maximum Likelihood method with bootstrap analysis of 1000 replicates. The obtained 16S rRNA sequences were deposited in the GeneBank under the following accession numbers: KX085478, KX085479, KX085480 and KX085481.

## Figures and Tables

**Table 1:** Sestonic microcystin concentrations during cyanobacterial bloom. (+) refer to the presence of *mcy*E gene. Errors represent standard error of two replicates.

Date	Sestonic MCs (µg·L^−1^)				*mcy*E
	MC-LR	MC-RR	MC-YR	Total MCs	
7 August 2012	0.31 ± 0.20	0.16 ± 0.02	0.07 ± 0.00	0.54 ± 0.22	+
4 September 2012	10.17 ± 1.59	3.14 ± 0.18	0.99 ± 0.07	14.31 ±1.84	+
24 September 2012	25.75 ± 6.10	16.82 ± 3.82	6.95 ± 1.45	49.52 ± 11.37	+
8 October 2012	13.64 ± 1.43	5.89 ± 0.74	2.42 ± 0.26	21.95 ± 0.43	+
29 October 2012	0.85 ± 0.26	0.52 ± 0.14	0.23 ± 0.06	1.60 ± 0.06	+

**Table 2:** Total microcystin concentrations in the water from different sampling dates enriched with 1.2 mg equivalent MC-LR·L^−1^ of crude MC extract before and after 15 days of incubation. Controls represent autoclaved water. Errors represent standard errors of two replicates. “n.d.” means “not detected”.

Date	Control (µg·L^−1^)		Reservoir Water (µg·L^−1^)	
	Day 0	Day 15	Day 0	Day 15
7 August 2012	1315 ± 32	1239 ± 26	1196 ± 80	n.d.
4 September 2012	1238 ± 35	1219 ± 95	1206 ± 22	n.d.
24 September 2012	1303 ± 15	1321 ± 52	1200 ± 30	n.d.
8 October 2012	1145 ± 69	1179 ± 125	1173 ± 65	n.d.
29 October 2012	1183 ± 45	1284 ± 22	1211 ± 44	n.d.

**Table 3:** Microcystin degradation rates under different media with variable total organic carbon and total nitrogen concentrations. Ratios correspond to assay period until 90% of total MCs degradation was achieved. (+) and (−) mean presence and absence of *mlr* genes, respectively. Errors represent standard errors of two replicates.

Bacterial Strains	*mlr*A-D Genes	Degradation Rates (µg MC L^−1^ h^−1^)		
		MSM	¼ R2A Medium	Reservoir Water
Y2	+	73.24 ± 6.09	113.36 ± 22.33	48.2 ± 16.80
2C20	−	12.75 ± 2.93	8.04 ± 4.94	20.62 ± 4.00
IM-1	+	171.15 ± 15.34	146.92 ± 23.79	144.9 ± 54.02
IM-2	+	25.35 ± 6.04	97.03 ± 40.47	11.39 ± 5.28
IM-3	+	37.10 ± 7.20	112.14 ± 21.36	16.30 ± 0.71
IM-4	−	19.42 ± 1.12	4.12 ± 1.37	21.26 ± 3.36

## Supplementary Materials

The following are available online at www.mdpi.com/2072-6651/8/11/318/s1, Table S1: Average bacterial growth rates (mg·L^−1^·h^−1^) during MCs degradation in MSM, reservoir water and ¼ R2A medium enriched with 1 mg equivalent MC-LR·L^−1^ of total MCs. Errors represent standard errors of two replicates. “n.d.” means not detected, Figure S1: Biodegradation of MC-LR and MC-RR variants by bacterial genotypes *mlr*^+^ (strains Y2, IM-1, IM2 and IM-3) and *mlr*^−^ (strains 2C20 and IM-4) incubated in MSM for 120 h. A negative control without bacteria was included. Error bars represent standard errors of two replicates, Figure S2: Biodegradation of MC-LR and MC-RR variants by bacterial genotypes *mlr*^+^ (strains Y2, IM-1, IM2 and IM-3) and *mlr*^−^ (strains 2C20 and IM-4) incubated in ¼ R2A medium for 120 h. A negative control without bacteria was included. Error bars represent standard errors of two replicates, Figure S3: Biodegradation of MC-LR and MC-RR variants by bacterial genotypes *mlr*^+^ (strains Y2, IM-1, IM2 and IM-3) and *mlr*^−^ (strains 2C20 and IM-4) incubated in reservoir water for 120 h. A negative control without bacteria was included. Error bars represent standard errors of two replicates.

## Abbreviations

The following abbreviations are used in this manuscript:

MCs	microcystins
*mlr*A^+^	presence of *mlr*A gene
*mlr*A^−^	absence (or no PCR amplification) of *mlr*A gene
*mlr*^+^	presence of the complete *mlr* gene cluster
*mlr*^−^	absence (or no PCR amplification) of the complete *mlr* gene cluster
TOC	Total Organic Carbon
TN	Total Nitrogen
PCR	Polymerase Chain Reaction
NCBI	National Center for Biotechnology Information
BLASTN	Basic Local Alignment Search Tool for Nucleotide
CyanoHAB	Cyanobacterial Harmful Algal Bloom
