# Supplementary material for: Presence or Absence of mlr Genes and Nutrient Concentrations Co-Determine the Microcystin Biodegradation Efficiency of a Natural Bacterial Community
Source: Toxins (Basel). 2016 Nov 3;8(11):318. doi: 10.3390/toxins8110318 (PMC5127115; doi:10.3390/toxins8110318)
Supplement: Supplementary file 1 [file toxins-08-00318-s001.pdf]

Supplementary Materials: Presence or Absence of *mlr* Genes and Nutrient Concentrations Co-Determine the Microcystin Biodegradation Efficiency of a Natural Bacterial Community

María Ángeles Lezcano, Jesús Morón-López, Ramsy Agha, Isabel López-Heras, Leonor Nozal, Antonio Quesada and Rehab El-Shehawy

Table S1. Average bacterial growth rates ($mg \cdot L^{-1} \cdot h^{-1}$) during MCs degradation in MSM, reservoir water and ¼ R2A medium enriched with 1 mg equivalent MC-LR·L^{-1} of total MCs. Errors represent standard errors of two replicates. "n.d." means not detected.

**Average bacterial growth rates ($mg \cdot L^{-1} \cdot h^{-1}$)**			
Bacterial strains	MSM	¼ R2A medium	Reservoir water
Control	n.d.	n.d.	n.d.
Y2	n.d.	0.25 ± 0.01	n.d.
2C20	n.d.	0.28 ± 0.00	n.d.
IM-1	n.d.	0.47 ± 0.00	n.d.
IM-2	n.d.	0.87 ± 0.06	n.d.
IM-3	n.d.	0.73 ± 0.04	n.d.
IM-4	n.d.	0.21 ± 0.01	n.d.

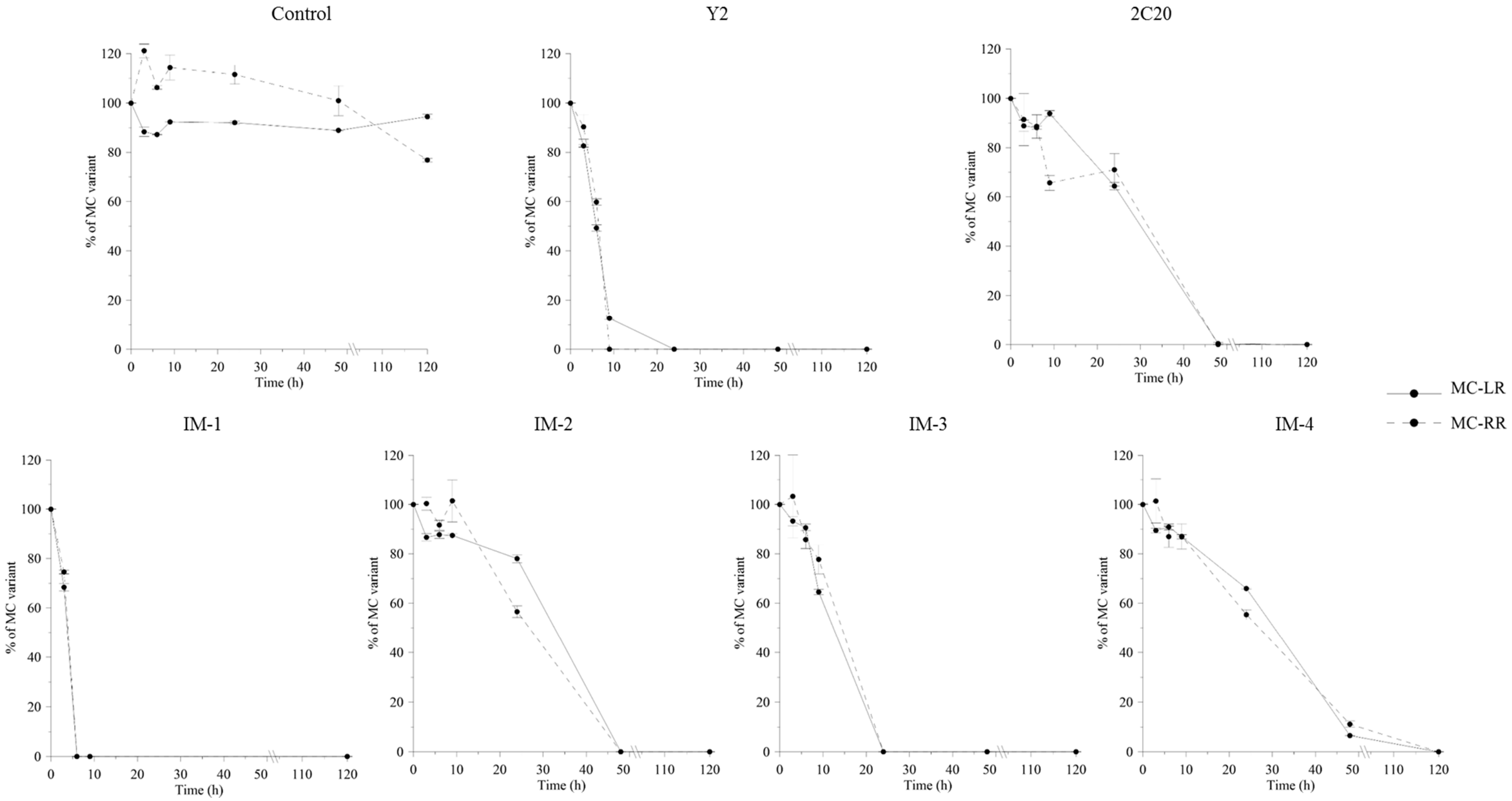

Figure S1. Biodegradation of MC-LR and MC-RR variants by bacterial genotypes *mlr*+ (strains Y2, IM-1, IM2 and IM-3) and *mlr*− (strains 2C20 and IM-4) incubated in MSM for 120 h. A negative control without bacteria was included. Error bars represent standard errors of two replicates.

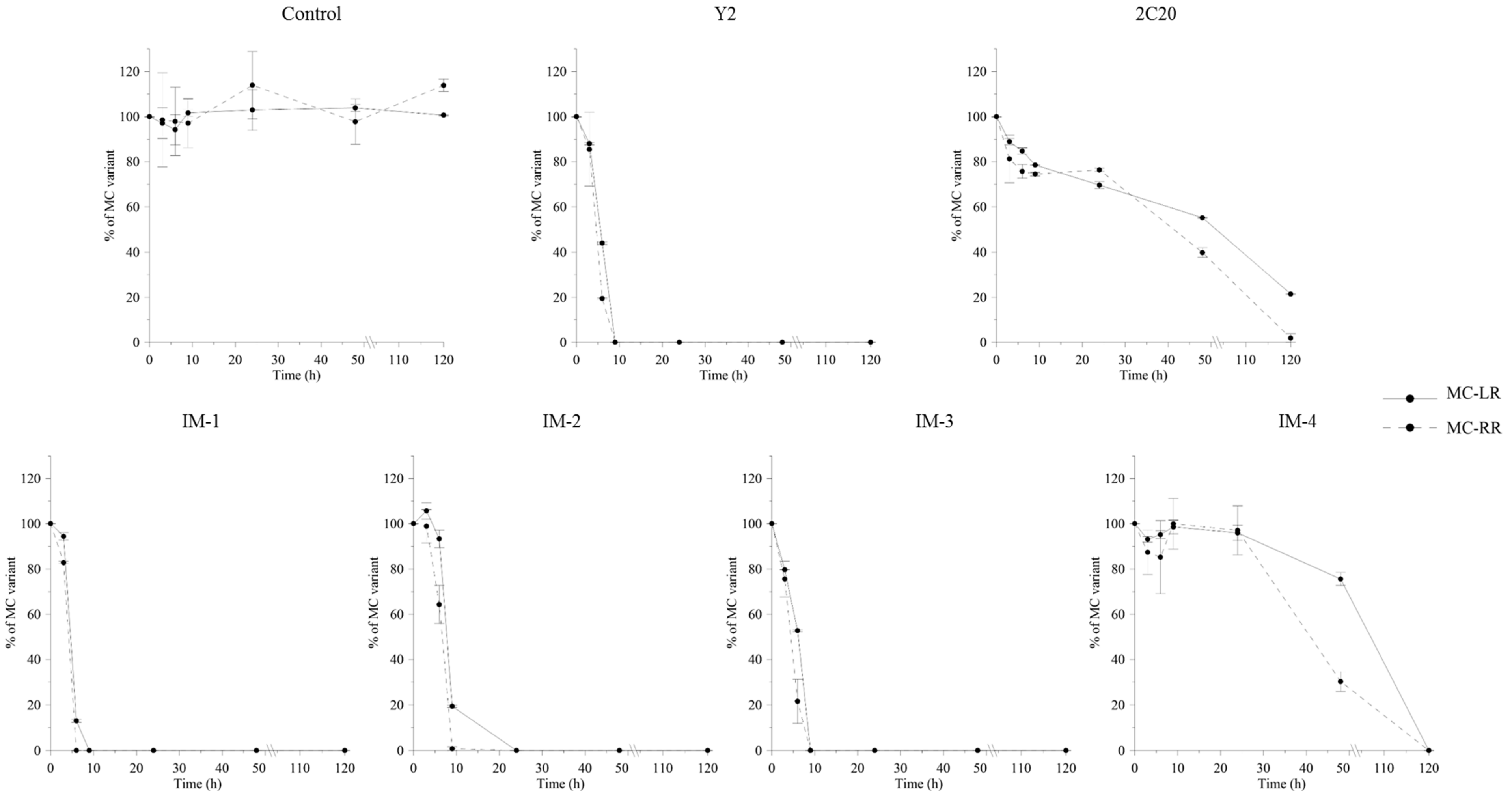

Figure S2. Biodegradation of MC-LR and MC-RR variants by bacterial genotypes *mlr*+ (strains Y2, IM-1, IM2 and IM-3) and *mlr*− (strains 2C20 and IM-4) incubated in ¼ R2A medium for 120 h. A negative control without bacteria was included. Error bars represent standard errors of two replicates.

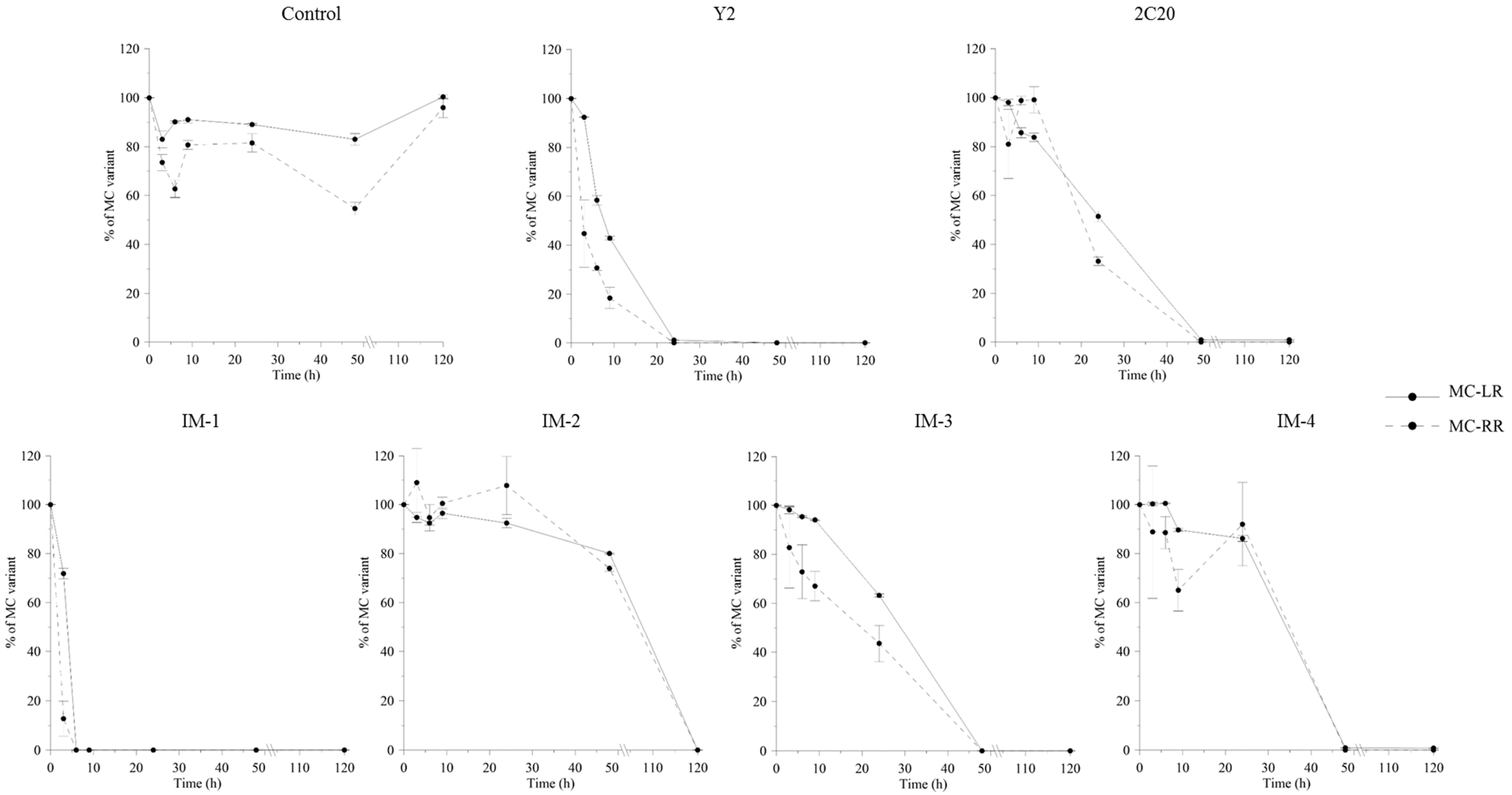

Figure S3. Biodegradation of MC-LR and MC-RR variants by bacterial genotypes *mlr*⁺ (strains Y2, IM-1, IM2 and IM-3) and *mlr*⁻ (strains 2C20 and IM-4) incubated in reservoir water for 120 h. A negative control without bacteria was included. Error bars represent standard errors of two replicates.
